# In the Pursuit of Green in COVID-19: Harnessing the Existing Talent to Pursue Green Corporate Entrepreneurship

**DOI:** 10.3389/fpsyg.2021.751961

**Published:** 2021-10-25

**Authors:** Essa Khan, Muhammad Shujaat Mubarik, Zubair Ali Shahid

**Affiliations:** ^1^Faculty of Business Administration and Social Sciences, Mohammad Ali Jinnah University, Karachi, Pakistan; ^2^Institute of Business Management, College of Business Management, Karachi, Pakistan

**Keywords:** talent management, green corporate entrepreneurship, organizational engagement, COVID-19, entrepreneurship

## Abstract

The study was conducted to ascertain the role of talent management practices in promoting green corporate entrepreneurship through the median of organizational engagement in the context of COVID-19. The study is quantitative and deductive in nature. The data was collected from 323 employees working in the large manufacturing industry. The data was collected through a self-administered survey and the data analysis was done through Smart-PLS, both measurement and structural models were evaluated. The study found that talent management is related to green corporate entrepreneurship. The study also confirmed the mediating role of organizational engagement relating talent management with green corporate entrepreneurship. This study will develop insight for the corporate managers and decision-makers to understand the intricacies of the talent management process and its interplay with green corporate entrepreneurship, and organizational engagement. Talent Management is concerned with the process of hiring, developing, and retaining highly competent incumbents for pivotal positions. Hence, the role of the talent management process invariably affects the organizational processes and outcomes like creativity and innovation, which come under the umbrella of corporate entrepreneurship. This study will not only add value in empirical research of the chosen concepts and constructs, but it will also bridge the theoretical gap between talent management and corporate entrepreneurship. It is the first study that related talent management practices to green corporate entrepreneurship. Furthermore, it is the first study that examined the mediating role of organizational engagement relating talent management to green corporate entrepreneurship.

## Introduction

Increased competition coupled with heightened environmental concerns has changed the dynamics of business. Firms, to survive and grow, are inexorably required to be competitive and environmentally friendly (Martinez-Martinez et al., 2019; Khan et al., 2021b). To achieve these complementing ends of competitiveness and environmental friendliness, firms, in future, need to have new ventures that along with being economically feasible are also required to be environmentally friendly. The need for environmental friendliness is further augmented with the rise of current pandemic known as COVID-19. COVID-19 has compelled the organizations to have an increased concern for employees along with being environmentally sustainable. The need for employee focus is augmented because of the pandemic caused job related uncertainty. Moreover, being environmental friendly is also a solution to COVID (Abubakar, 2020; Dwivedi et al., 2020; Maritz et al., 2020). The existing concept of corporate entrepreneurship, defined as activities aimed at creating new businesses in the established companies (Vanacker et al., 2017), addresses the competitiveness of the firm, but does not address the environmental concerns. Such a strategy, despite its sound track (Kuratko, 2017), does not bode well with the more environmentally aware and active customers. So, the way forward is sustainable or green corporate entrepreneurship, the genre of corporate entrepreneurship fusing the innovativeness of corporate entrepreneurship with the idea of sustainability (Miles et al., 2009). Sustainable corporate entrepreneurship along with the traditional innovation in business model, includes; responsible environmental management, social accountability and economic performance (Miles et al., 2009).

The predecessor of green corporate entrepreneurship has been studied for its possible determinants, linking mechanisms and boundary conditions. The search for the determinants resulted in the following list; knowledge management (Guadamillas et al., 2008), human resource practices (Hayton, 2005; Kühn et al., 2016), high-performance human resource practices (Zhang et al., 2008; Shehata et al., 2020). Additionally, the academicians, exploring the linking mechanism relating corporate entrepreneurship relating to different determinants, found organizational citizenship behavior (Zhang et al., 2008), knowledge behavior (Mustafa et al., 2013) and absorptive capacity (Shafique and Kalyar, 2018). On the other hand, the understanding of sustainable corporate entrepreneurship is at nascent stage. A study conducted in South Africa found organization's environmental concern to be related to environmentally responsible intrapreneurship (Christos, 2017). Despite being the only way forward, the absence of scholarly work on green corporate entrepreneurship is an encouraging sign. The current study is being undertaken to fill the existing void.

The strong ripples and undercurrents created by the hyper-competitive world spares no one (Khan et al., 2020). Business giants like Blackberry, Kodak, and Panasonic were once household names, are now unknown entities for the new generation. Despite the severe competition, some firms have maintained their existence to this day. Apple and Google are the two names that have successfully managed to ward off the annihilating prowess of competition (Denning, 2021). It raises the question as to what differentiates between the firms that survive and grow in the face of the ever-changing world caused by newly emerging ideas (Sharon, 2020). The answer to this moot is the ability of the firms to search and use the emerging opportunities (Jiang et al., 2021). The effectiveness of corporate entrepreneurship in improving the firm performance has catapulted an increased interest by the researchers. Different ways are being evaluated for their usefulness in affecting corporate entrepreneurship.

The role of human resource management is pivotal in motivating the employees to indulge in extra-role behavior (Bos-Nehles and Veenendaal, 2019). In the same vein, HR practices can be attributed to green corporate entrepreneurship (Gardas et al., 2019). One of the main components of sustainable HRM is to manage the talent (Ehnert, 2011). Talent management; the acquiring talent, developing and retaining talent (Collings et al., 2019), is being studied for its role to instigate green corporate entrepreneurship. In the preceding lines, an attempt is being made to explicate how talent management practices can be instrumental in harnessing the creative potential of employees to indulge in green corporate entrepreneurship. Two reasons are being forwarded for the appropriateness of talent management to affect green corporate entrepreneurship. First, talent management practices groom the employees to have a broad perspective along with positive work attitude and organizational congruence (Mensah, 2015; Mensah, 2019). With the augmented resourcefulness and improved understanding, employees work for sustaining the organization along with sustaining the environment as talent management is found to be related to innovation (Salau et al., 2018; van den Broek et al., 2018) and sustainable work behavior (Mujtaba and Mubarik, 2021). In light of this evidence, a positive relationship between talent management practices and green corporate entrepreneurship is proposed. Second, the employee centered practices of talent management practices provide the employees with the impetus to reciprocate in the same coin. Using the basic tent of social exchange theory (Blau, 1964), we propose that employee centric organizational practices will invoke similar behavior among the employees. Green corporate entrepreneurship is one such pro-organizational behavior expected from the employees. In this study, an attempt is being made to ascertain the role of talent management to increase green corporate entrepreneurship. Second, the study is seeking to unfurl the mediating role of organizational engagement linking talent management practices with green corporate entrepreneurship.

## Literature Review

### Sustainable Corporate Entrepreneurship

Corporate entrepreneurship is one of the critical strategic approaches in this globally competitive economy. Corporate entrepreneurship is also found to work as a change agent for the community (Martín-Rojas et al., 2020). In recent times organizations are highly motivated to become more entrepreneurial in their practices and approaches (Peschl et al., 2020; Shehata et al., 2020). Creativity and Innovation are mostly dependent on entrepreneurial activities and individual mindsets. Employees with talent are considered to be a source of ideas, intellect, and innovation (Sadat and Nasrat, 2020). The fruition of corporate entrepreneurship is linked with the presence and use of talented individuals who are capable of generating new ideas and going to the process of creativity and innovation (Kabir, 2019). To some researchers corporate entrepreneurship to be a booster for firm profitability and economic development (Mayer and Motoyama, 2020; Si et al., 2020).

The rising concern for environment has evolved the business thinking. Instead of parochially concentrating on entrepreneurial performance, the firms, realizing the importance of environment, have begun to incorporate sustainability in their ideas. The ensuing sustainable corporate entrepreneurship is the embodiment of businesses that value both sustainability and corporate entrepreneurship (Provasnek et al., 2017). The slack at either bleeds the firm differently. The firm, being sustainable but not entrepreneurial, will not be economically feasible. On the other hand, being entrepreneurial but uncaring toward the environment, damages firm's standing with the customers (Provasnek et al., 2017). Against this back drop, the concept of sustainable corporate entrepreneurship can ensure the safe sailing of the firms. Sustainable corporate entrepreneurship is achieved when a firm along with attaining innovation in products, processes and strategies also achieve three sustainability goals of; responsible environmental management, social responsibility and economic performance (Miles et al., 2009). Large organizations need more innovative and creative products and processes to sustain and to get a competitive edge. Large scale manufacturing sector also focuses on developing new products, services, processes, technology, administrative techniques, techniques, practice, and competitive positions (ur Rahman, 2019; Thompson et al., 2020).

### Talent Management

The war for talent and management of talent remain major challenges for organizations. Talent familiarity with fourth industrial revolution (4IR) advancement is needed to foster environmental sustainability (Bamel et al., 2020; Farndale et al., 2020; Ogbeibu et al., 2021). Environmental sustainability is a competitive advantage for contemporary organizations and that is why most of the organizations are willing to gain it (Ab Wahab, 2021; Atiku and Fapohunda, 2021). Besides, talent is considered a scarce, strategic asset and a source of competitive advantage (Harsch and Festing, 2020). Stemming from the resource-based view (RBV), skills may be understood as valuable, uncommon, inimitable, and non-substitutable, allowing the implementation of value-creating strategies and success of sustainable competitive advantage (Sparrow and Makram, 2015; Chadwick and Flinchbaugh, 2021).

The realization of employees as a source of competitive edge has given a strategic outlook to HR. Instead of managing day to day staffing through recruitment and training, HR was entrusted to ensure competitive advantage of the firm by attracting, hiring, and retaining the talented individuals. The effort to define talent management is preceded by the definition of talent itself. The talented individuals have mastery of the skills that place them among the top 10% of the employees (Gagné, 2000). Along with their knowledge, skills, and competencies, they have a positive attitude toward their work (Tansley, 2011). In simple words, talent management is the management of talented individuals. But such a simplistic definitions hide more than it reveals. So, a comprehensive definition is required. Initially, talent management was considered to be grooming the talented ones and exiting the ones lacking in talent (Michaels et al., 2001). However, the use of talent management focusing on employees instead of position may not be theoretically sound. So, to improve the theoretical soundness of the definition, another definition focusing on position rather than person has been forwarded. According to this definition talent management is the systematic identification of key positions, development of a talent pool for the identified positions, development of the HR architect to fill these positions and finally ensuring the incumbents continued commitment to the organization (Collings and Mellahi, 2009; Collings et al., 2019). Talent management is found to be related to organizational performance (Ahmad Arif and Uddin, 2016).

### Organizational Engagement

At personal level, work engagement is defined as a positive, fulfilling, work-related state of mind characterized by vigor, dedication and absorption (Bakker and Leiter, 2010; Schaufeli, 2012). As feelings are contagious therefore employees, finding their colleagues to be imbued with work engagement, catch the bug by interpreting and ascribing the collective meaning to prevailing work behavior (Seibert et al., 2004). So, building upon the personal level work engagement, organizational engagement has been defined as the shared perception of employees regarding their collective physical, cognitive and emotional involvement in their work (Barrick et al., 2015). As a collective resource for the firms, organizational engagement affects organizational performance (Barrick et al., 2015; Schneider et al., 2018).

Organizational members have a shared perception that members of the organization are engaged in their work (Khan et al., 2021a). Collective organizational engagement is an understudied topic for researchers and can help firms achieve and sustain higher performance (Barrick et al., 2015). To Trabucchi et al. (2020) organizational engagement is fundamental to motivate employees and their involement in innovation (Trabucchi et al., 2020). This positive state of mind brings positive attitudinal, behavioral, and work-related results among the employees (Saks, 2021). As a result, employees are willing to allocate extra time and resources to their organizations (Lee et al., 2018).

### Talent Management and Green Corporate Entrepreneurship

Both the current performance of the firm and its future prospects are affected by its employees and their attitude (Long, 1980; Berberoglu, 2018). Firms with employees who are competent and willing can do wonder for their organizations. Talent management, from hiring to facilitating the employees, has the potential to affect green corporate entrepreneurship. Hiring the competent individuals desiring to work for the sustainable performance of the firm, and training them to further improve their competence to pursue green corporate entrepreneurship work to enable the organization to achieve this end. Corporate entrepreneurship is the organizational-led creative effort that emanates from the employees of the organization (Amberg and McGaughey, 2019). The creativity component, in any work, brings the uncertainty causing risk into it. Employees tend to avoid all those enterprises related risk because of the associated negative repercussions (Ratten, 2020). In such a situation, spurring employees to indulge in any risky enterprise is possible when employees feel that they are being actively nurtured and supported (Astrini et al., 2020). The current study purports that talent management practices ensure the employees of the support and equip them with the required resources to pursue corporate entrepreneurship. When an organization is caring, valuing, and supporting its employees, inundated with such respect, the employees in return build a trusting relationship with the organization. (Al Hammadi et al., 2020). The employees feel that such caring and supportive behavior readily indulges them even in a risky enterprise (Al Hammadi et al., 2020). In addition, organizations are applying talent management practices to equip employees with the required resources to accomplish innovation results (Sopiah et al., 2020). Employees, finding the organization to provide them with the required training and extending other exposures to enhance their performance, have the necessary skills and resources to invigorate their efforts to accomplish the goal of corporate entrepreneurship (Othman and Khalil, 2018).

Additionally, talent management is a process that works for the development of employees (Collings et al., 2015). The resulting positive benefits accrued to the employees work as favors extended to them. According to social exchange theory (SET), (Blau, 1964), individuals seek the opportunities to return the favor in the same currency. So, the obliged employees show their proclivity to indulge in activities valued by the organization (Blau, 1964). Enabling the firm to sustainably operate is one of the ways employees can return the favor extended to them by the organization. Due to its conceptual similarity with the above-mentioned constructs, corporate entrepreneurship can be expected to be influenced by talent management practices. In light of the existing theoretical underpinning and empirical evidence, the following hypothesis can be formed. So, in the light of SET, we can have the following hypothesis.

*H1: Talent management is related to green corporate entrepreneurship*.

### Talent Management and Organizational Engagement

The relation between talent management and organizational engagement can be explained in three ways. First, the idea of talent management gives value to the employees (Murillo and King, 2019). Apart from hiring that is one time process, the other facets of talent management such as development and retention of employees continually work to cater to the needs of the employees (Collings et al., 2019). Furthermore, talent management works for the growth of the employees (Boštjančič and Slana, 2018). Finding themselves being facilitated and groomed, the employees value their work and organization positively and show increased level of absorption in their work in the context of the organization (Deery and Jago, 2015). Second, the process of talent management works as a favor extended to the employees (De Boeck et al., 2018). In return, the favored employees seek opportunities to return the favor. One of the ways to positively reciprocate the favor is to show enhanced organizational engagement (Blau, 1964). Third, talent management works as a resource (Luna-Arocas et al., 2020). Employees who are resourceful have positive energy and they show more engagement both to their work and organization. Though talent management has not yet been related to organizational engagement, supportive HR practices, a facet of talent management, has been found to be related to employee engagement (Juhdi et al., 2013).

Talent management, on part of the organization, is the augmented care for the employees (Claus, 2019). Through talent management practices, firms strive to provide employees with extra care to retain them and provide them with training to enhance their skills and abilities (Cross Walker, 2020). The talent management practices, from the identification of key posts to whole-hearted efforts to retain the employees, strive to facilitate employees by providing them with care, learning opportunities, and resources. Such a caring attitude on the part of an organization is enough to overwhelm the employees (Hamilton and Davison, 2018) and they seek opportunities to return the favor. There are multiple ways for employees to return the favor. Employees can put more effort into their work. Employees can show more commitment toward the organization. In line with the above-mentioned constructs, employees can be highly engaged with the organization. According to social exchange theory, employees served by the organization will attempt to return the favor. One way to return the favor is to increase their organizational engagement (Blau, 1964). Researchers have found that employees have a heightened organizational engagement when firms are supportive to employees (Saks, 2021). Additionally, organizational engagement increases with improved HR practices (Afsar et al., 2020). Talent management is also a type of enhanced human resource. Therefore, the same effect of increased employee engagement can be attributed to talent management practices (Sopiah et al., 2020). Building upon the aforementioned discussion, the following hypothesis is proposed.

*H2: Talent management is related to organizational engagement*.

### Organizational Engagement and Green Corporate Entrepreneurship

Engagement is a state characterized with pleasure and activation (Bakker and Oerlemans, 2011). Collectively, pleasure and activation give energy and drive to employees to pursue organization's objectives (Khan et al., 2021a). Organizational engagement is a positive state in which employees are not only ready to be actively involved in their work, but they also work for the goals of the organization. One of the pursued goals of the organization today is green corporate entrepreneurship (Miles et al., 2009). As mentioned earlier, organizationally engaged employees also engage them with the goals of the organization and actively pursue them, so it is proposed that organizational engagement will have effect on green corporate entrepreneurship.

One of the major purposes of strategies is to ensure the sustainability of a firm (Parente et al., 2020). A firm that can use its resources to meet the customers' current needs and plan for meeting their future needs can survive in the long run (Takata et al., 2020). Because of the uncertain future, firms are concerned about the business ideas that can cater to the customers' rising needs (Atiku and Abiola, 2020). Corporate entrepreneurship is one of the strategies to serve this purpose. Firms have realized that corporate entrepreneurship is employee-driven. Employees with their accumulated knowledge are in a position to indulge in CE (Belousova et al., 2020). Google uses its employees for this purpose. The moot point is why some organizations can use their employees to pursue corporate entrepreneurship while others cannot. One of the possible explanations lies in the organizational engagement of the employees. Firms with engaged employees have positive emotions so they both can reciprocally benefit each other (Pandey et al., 2020). Employee engagement is consist of two mechanism: attention (intellectually available and time given to a role) and concentration (to involve in a role to the best of his/her ability), (Peng et al., 2014). The presence of positive emotions increases the thought-action repertoire of the employees that result in an increased number of ideas and enables employees to vigorously follow their implementation, resulting in an increased number of corporate entrepreneurship activities (Belousova et al., 2020). Currently, there is no study that has explored the relation between organizational engagement and corporate entrepreneurship. In the light of the above discussed reasoning and empirical evidence, the following hypothesis is formed.

*H3: Organizational engagement and green corporate entrepreneurship*.

### Mediating Role of Organizational Engagement

Trabucchi et al. argue that in this global competitive environment businesses have to improve productivity and one of the ways to do this is through employees. There is evidence that engaged employees are more, loyal, trusted, hard worker, and they are ready to go the “extra mile” for their organization. Employee engagement is linked with organization performance and employees are more enthusiastic about their jobs (Trabucchi et al., 2020). If the values and norms of an individual are associated with organization, then synergy of organization and employee creates engagement resultantly reciprocal beneficial for employee and organization outcome (Afsar et al., 2020). The social exchange theory (Blau, 1964) explains that in a mutual bond, if an employee finds connected and well-fit with the organization and its values, norms and standards, then the employee finds well-fit with the organization an employment. This validates the importance of developing employee engagement strategies for organizations and organizations be able to develop their sustainable competitive advantage through their talent (Nienaber, 2019).

Despite the well-represented theoretical understanding between talent management and corporate entrepreneurship and talent management and organizational engagement, the role of organizational engagement as a mediator between talent management and corporate entrepreneurship requires further explanation. Research also proves that talent management is positively related to organizational performance (Mohammed, 2016; Bakytgul et al., 2019), employee retention (Pandita and Ray, 2018), and corporate entrepreneurship strategies (Ratten and Ferreira, 2016). Employees' engagement is playing a pivotal role in the development of organizations (Bakytgul et al., 2019). Employees' engagement increases organizational engagement and that ultimately results in increased organizational performance and job satisfaction (Rai and Maheshwari, 2020). Based on the social exchange theory (Blau, 1964), employees with talent are supported by organizations and keep them engaged and motivated. In return, the employees are coming up with creative and innovative ideas that can add value to organizations' products or services (Afsar et al., 2020).

Social exchange theory posits that an extended favor brings back a favor (Blau, 1964). Employees working in the organization regard their firm to be a personified entity and a favor extended to them is regarded a benevolence by the firm. The practices of talented management directed toward employees are meant, on one hand, to facilitate the employees (Ambrosius, 2018). On the other hand, the talent management practices groom the employees to improve their skills and knowledge of the work (Luna-Arocas et al., 2020). Employees regard both the aspects of talent management to be favors extended to them, and in return seek opportunities to reciprocate. One of the ways available to employees to reciprocate is to show more engagement in their work. The individual work engagement is the building block of collective organizational engagement. Pleased with the organizational policies toward them, employees begin to demonstrate high level of organizational engagement (Rofcanin et al., 2017). The engaged employees strive to serve their organization in multiple ways. Apart from being efficient in their work (Rofcanin et al., 2017), they search for new ventures ensuring the growth of their firm. Aware of the increased prominence of environmental concern, the employees, seeking to return the favor, indulge in corporate entrepreneurial activities that are environmentally friendly. Building upon the mentioned reasoning, the following hypothesis is formed.

**Figure 1:**
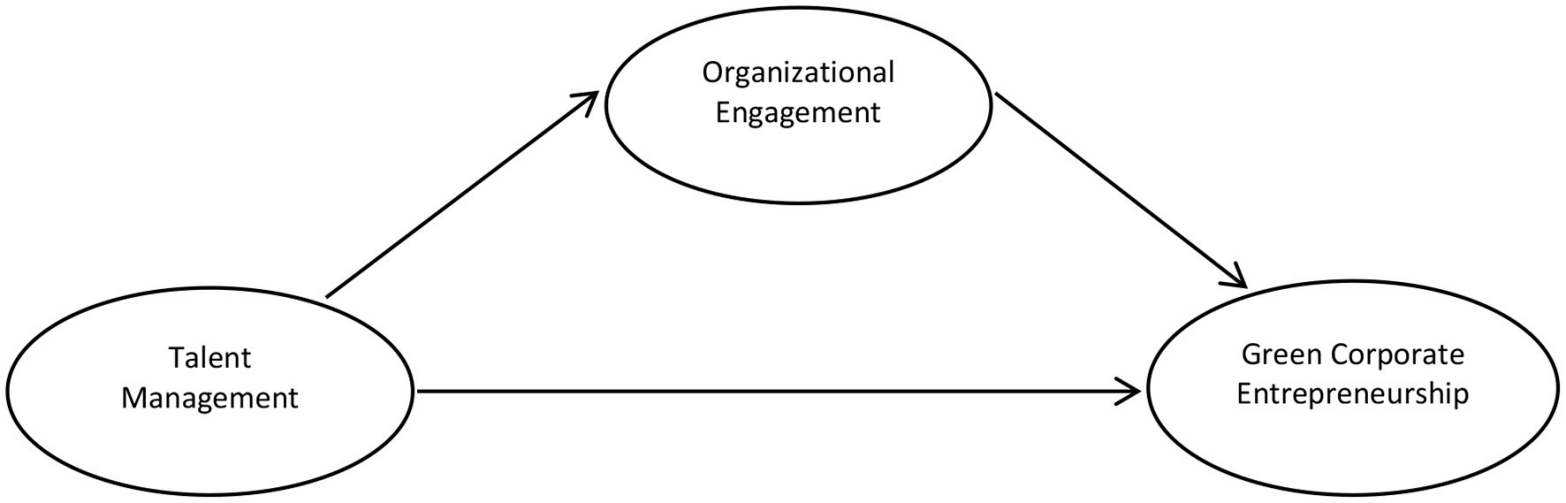
Conceptual framework of the study.

*H4: Organizational engagement mediates the relationship between talent management and green corporate entrepreneurship (**Figure 1**)*.

## Methodology

### Sampling and Data Collection

The study collected data from the individuals working in the large manufacturing firms of Pakistan. Initially, 400 potential respondents were approached from the manufacturing industry. The approached individuals were the ones working in the large manufacturing firms of the country. Out of the approached 400 employees, 323 responded and filled the questionnaire. The overall response rate is 81%. The profile of the respondents is given in Table 1. The majority of the respondents were male (79%) while the remaining 21% were female as shown in Table 1. Moreover, Table 1 shows that a small portion of 7% of respondents had 12 years (Intermediate) of qualification, besides this 38% of the respondents had bachelor degree and the remaining 54% were master degree holders. A larger portion of respondents came from line managers (48%), while the portions of middle and top managers were 35 and 15% respectively. Finally, Table 1 shows that the average age of the respondents was 33.37 years and their job experience was 9.47 years.

**Table 1:** Respondents profile.

**Variable**		***n* (323)**	
Gender	Male	79%	
	Female	21%	
Qualification	Intermediate	07%	
	Bachelor	38%	
	Master	54%	
Management level	Line manager	48%	
	Middle manager	35%	
	Top manager	15%	
		**Mean**	**SD**
Age		33.37	9.256
Experience		9.47	8.525

### Measure

#### Talent Management

To measure talent management, the scale developed by Alkerdawy (2016) was adopted (Alkerdawy, 2016). The scale has 18 items that were measured on five points Likert scale where one means strongly disagree and five means strongly agree. One of the example items of talent management scale is: “Our organization attracts talent effectively.”

#### Green Corporate Entrepreneurship

To measure green corporate entrepreneurship, the study adapted the scale developed by Zahra (Zahra, 1996). The original scale was meant to measure corporate entrepreneurship. For this study, the items of the scale were adapted to measure green corporate entrepreneurship. One of the adapted item is as follows: “Our organization has pioneered sustainable innovation in the industry.” All the items were measured on five points Likert scale where one was used to denote “strongly disagree” and seven was used to denote “strongly agree.”

#### Organizational Engagement

The organizational engagement scale is developed by Barrick et al. (2015). The scale has 0.6 items measured on Likert scale where one stands for strongly agree whereas five represents strongly agree. One of the example items of organizational engagement is “Employees in our organization really throw themselves into their work.”

## Findings

### Measurement Model

This study used PLS-SEM to analyze the collected data. PLS-SEM has been widely used in a variety of fields in recent years with non-normal data, small sample sizes and the use of formative indicators being the most prominent reasons for its application (Hair et al., 2019). The popularity of structural equation modeling (SEM) has grown out of the need to test complete theories and concepts (Hair et al., 2014). According to Hair et al., currently covariance-based structural equation modeling (CB-SEM) is the dominant method for analyzing complex interrelationships between observed variables and latent variables (Hair et al., 2019). Much of SEM's success can be attributed to the method's ability to evaluate the measurement of latent variables, while also testing relationships between latent variables (Sarstedt et al., 2014). Moreover, reliability and validity are the two most important criteria used in PLS-SEM analysis to evaluate the outer model (Hair et al., 2014). Reliability is assessed using composite reliability (CR) while validity is measured through convergent validity average variance extracted (AVE), and discriminant validity using Fornell and Larcker (1981) criterion and indicator's outer loadings.

The constructs used in the study were evaluated for their reliability and validity. Reliability is a measure of consistency, and the measures used were Cronbach's alpha and composite reliability. The minimum acceptable level for both the measures is 0.7 (Holmes-Smith et al., 2006). Validity indicates the ability the measure what it aims to measure. Convergent and discriminant validity were checked at the item as well as at the construct level. Item loading was used for item-level convergent validity, and the minimum value for item loading is 0.7. AVE (Average Variance Extracted) was used for construct level validity, and the minimum acceptable value of AVE is 0.5 (Fornell and Larcker, 1981; Henseler et al., 2009; Hair et al., 2010). Discriminant validity was also measured at the item and construct level, which is the ability of a construct to stand out from the different measures. For item level discriminant validity, the loadings are required to be more than cross-loadings (Fornell and Larcker, 1981; Bagozzi and Yi, 1988). Whereas, for construct level, both Fornell and Larcker and HTMT criterion are used.

The results contained in Table 2 shows the used constructs to be reliable and valid. The constructs used in the model namely; green corporate entrepreneurship, talent management and organizational engagement were found to be reliable as the Cronbach's Alpha (Alpha) and composite reliability (CR) were more than the minimum acceptable value of 0.7. The lowest value of alpha (0.897) and CR (0.921) were found for organizational engagement as shown in Table 2. Item level and construct level convergent validity were evaluated through the values of item loadings and average variance extracted (AVE). Though some items had loadings of less than the recommended threshold of 0.7, at the construct level AVEs were more than 0.5, the lower bound for the construct to have convergent validity. The lowest value of AVE was found for green corporate entrepreneurship (AVE = 0.503) as given in Table 2. Finally, to evaluate the constructs for the discriminant validity, hetero-trait mono-trait (HTMT) ratios were computed for the constructs used in the study. As shown in Table 3, all the ratios are <0.85, the stricter upper bound for the constructs to be declared different (Henseler et al., 2015).

**Table 2:** Reliability and validity.

**Construct**	**Items**	**GCE**	**OE**	**TM**	**TL**	**Alpha**	**CR**	**AVE**
Green corporate entrepreneurship	GCE1	0.781				**0.922**	**0.933**	**0.503**
	GCE2	0.82						
	GCE3	0.761						
	GCE4	0.791						
	GCE5	0.608						
	GCE6	0.618						
	GCE7	0.643						
	GCE8	0.735						
	GCE9	0.777						
	GCE10	0.519						
	GCE11	0.68						
	GCE12	0.688						
	GCE13	0.686						
	GCE14	0.753						
Organizational engagement	OE1		0.834			**0.897**	**0.921**	**0.661**
	OE2		0.851					
	OE3		0.818					
	OE4		0.847					
	OE5		0.708					
	OE6		0.811					
Talent management	TM1			0.764		**0.963**	**0.967**	**0.62**
	TM2			0.843				
	TM3			0.802				
	TM4			0.792				
	TM5			0.862				
	TM6			0.791				
	TM7			0.783				
	TM8			0.549				
	TM9			0.836				
	TM10			0.862				
	TM11			0.821				
	TM12			0.808				
	TM13			0.844				
	TM14			0.784				
	TM15			0.766				
	TM16			0.676				
	TM17			0.735				
	TM18			0.791				

**Table 3:** Descriptive statistics and discriminant validity.

			**Correlation**		**HTMT Ratios**	
**Variable**	**M**	**SD**	**(1)**	**(2)**	**(1)**	**(2)**
Organizational engagement (1)	3.822	0.855				
Green corporate entrepreneurship (2)	3.626	0.778	0.679^**^		0.749	
Talent management (3)	3.732	0.878	0.753^**^	0.790^**^	0.812	0.842

** *Correlation is significant at the 0.01 level (2-tailed)*.

### Structural Model

Testing the structural model is possible when the constructs used in the model are related. To evaluate this requirement, inter-constructs correlations were computed. The results, given in Table 3, demonstrate that the constructs are either moderately or strongly related; thus, paving the way for model testing being described here. The minimum value of correlation is found between organizational engagement and green corporate entrepreneurship (*r* = 0.679). The model proposed in the study has four hypotheses. The first hypothesis conjectured a relationship between talent management and green corporate entrepreneurship. The results obtained supported the claim (β = 0.669, *p* = 0.000). The second hypothesis, claiming a relationship between talent management and organizational engagement, was also found to be significant (β = 0.765, *p* = 0.000). Similarly, the third hypothesis purporting a relationship between organizational engagement and green corporate entrepreneurship turned out to be significant (β = 0.181, *p* = 0.002). Along with the reported three direction relationship tested, the study also explicated the mediating role of organizational engagement relating talent management with green corporate entrepreneurship. The results, shown in Table 4, provide support to the mediating role of organizational engagement (β = 0.139, *p* = 0.003).

**Table 4:** Structural model.

**Relation**	**Coefficient**	**SE**	***t*-test**	***p*-value**
Talent management GCE	0.669	0.049	13.634	0.000
Talent management Organizational engagement	0.765	0.028	27.761	0.000
Organizational engagement GCE	0.181	0.058	3.099	0.002
Talent management organizational engagement GCE	0.139	0.047	2.981	0.003
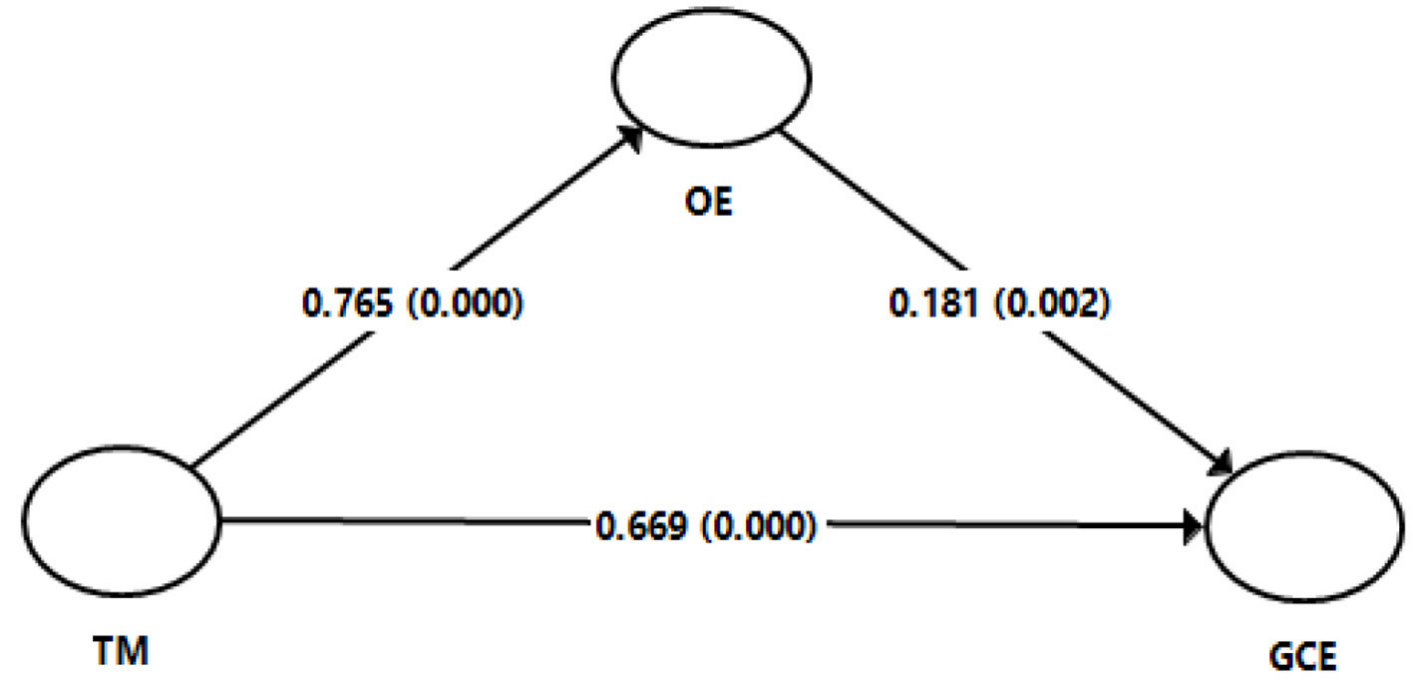				

*GCE, Green Corporate Entrepreneurship; TM, Talent Management; OE, Organizational Engagement; GCE, Green Corporate Entrepreneurship*.

## Discussion

The study was undertaken to explicate the role of talent management in affect green corporate entrepreneurship. Additionally, the study intended to fathom the mediating role of organizational engagement relating talent management with green corporate entrepreneurship. In total, the study tested four hypotheses. The first hypothesis, purporting a relationship between talent management and green corporate entrepreneurship, was found to be significant. This finding is in line with the earlier findings suggesting a relation between talent management and employee performance (Mensah, 2015; Choi and Cho, 2021). The second hypothesis delineated a relationship between talent management and organizational engagement. The obtained results showed the claim was supported in line with the earlier studies depicting a relationship between HR practices and organizational engagement (Juhdi et al., 2013). The findings of the study are being discussed in the subsequent paragraphs.

The first hypothesis proposed a relationship between talent management practices and green corporate entrepreneurship. The findings supported the conjuncture, which is in line with the findings of the earlier studies (Al Hammadi et al., 2020; Astrini et al., 2020; Ratten, 2020; Sopiah et al., 2020). Though no study has attempted to divulge the role of talent management practices in affecting green corporate entrepreneurship, earlier studies had pointed to the relation between talent management practices and innovative work behavior and creativity (Othman and Khalil, 2018). The finding of the current study corroborates with the earlier finding relating to talent management practices and creativity (Sopiah et al., 2020). The second hypothesis of the study proposed a relation between talent management practices and organizational engagement. Although there is a void of literature on the relationship between talent management practices and organizational engagement; some studies are exhibiting the relationship between a supportive organization and organizational engagement (De Boeck et al., 2018; Afsar et al., 2020; Luna-Arocas et al., 2020; Saks, 2021). The finding of the current study is in line with the findings of aforementioned researchers. The third hypothesis asserting a relationship between organizational engagement and green corporate entrepreneurship was also synched with the earlier studies that had found organizational engagement to be related to other organizational performances (Srivastava et al., 2014; Barrick et al., 2015; Kazimoto, 2016). Besides the above-discussed direction relationship, the study endeavored to unfurl the mediating role of organizational engagement in talent management and green corporate entrepreneurship. Though, there is no current study that has found such a mediating role for organizational engagement, the current study found organizational engagement to be mediating the relation.

Finally, talent management was related to organizational engagement in the light of existing evidence and theory. The final direct relation between talent management and green corporate entrepreneurship was found to be substantiated. Apart from their significant relation with green corporate entrepreneurship; talent management and green corporate entrepreneurship were related to organizational engagement. The findings showed that organizational engagement mediated the relationship between talent management and green corporate entrepreneurship; thus, affirming the role of positive emotions and resources. Talent management triggered engagement and innovativeness in employees pushes the employees to return the favor, as described in social exchange theory (Blau, 1964), by indulging in innovation behavior. Positive emotions and resources increased because indulgence in organizational engagement helps employees submit to corporate entrepreneurship.

## Theoretical Contribution

The current study makes two contributions to existing knowledge. First, from the lens of social exchange theory, individuals reciprocate to the favor extended to them. The practices of talent management are intended to groom and develop the employees. Responding to these practices and the associated benefits, employees are inclined to indulge in extra-role behavior meant to benefit the organization. Green corporate entrepreneurship is one such behaviors that are manifested by the employees in order to return the favor extended to them. Second, the study unfurled the mediating role of organizational engagement relating talent management practices to green corporate entrepreneurship. This finding is also in line with social exchange theory. Employees encouraged by the employee centered practices tend to reciprocate positively to the positive overtures of the firm and as a result show higher organizational engagement that leads organizational specific extra-role behavior such as green corporate entrepreneurship.

## Managerial Implications

The findings of the study has three managerial implications. First, the use of talent management can be made for giving a surge to organizational engagement. There is evidence suggesting organizational engagement improve organizational performance (Barrick et al., 2015; Bakytgul et al., 2019). Firms, by giving a push to organizational engagement can reap these benefits. In the context of COVID-19, the idea of stimulating organizational engagement becomes more important as the increased engagement will ensure employees' inclination to indulge in pro-organizational behavior. Second, the organizations can harness the creative potential of their employees through talent management practices. Attracting the talent, further developing it and facilitating it to pursue green corporate entrepreneurship. Third, organizations while further grooming the talented individuals can imbibe them with the rising importance of sustainability. As a result, employees with talent may consider environmental aspects of their innovative pursuit thus ensuring green corporate entrepreneurship.

## Limitation and Future Areas of Studies

Reflecting on a journey always reveals what could have been improved; the same is also true for the current study. At the end of the study, we find the study to be infected with some limitations. First, the study collected the data only from employees; the process is safe as long as it is regarding the practices of management of the behavior of the leadership, but data collection from employees regarding their performance such as corporate entrepreneurship is prone to self-reporting bias (MacKenzie and Podsakoff, 2012). Future studies can go for employee-manager dyads to reduce common method error. Second, the current study collected the data at a single point; such a procedure does not allow for the cause to register its effect. To improve the claim of causality, the studies in the future can collect data for cause and effects at two different times. Third, the current study has collected data from the large-scale manufacturing sector. The narrow focus limits the generalizability of the study. Researchers in future, studying the same relation, can widen the population to improve the generalizability of the findings.

## Data Availability Statement

The raw data supporting the conclusions of this article will be made available by the authors, without undue reservation.

## Author Contributions

The authors contributed to the study conception and design. Literature review, methodology, search of relevant articles, data collection, analysis, discussion, results, and conclusion were performed by EK and MM. The first draft of the manuscript was written by EK. MM and ZS thoroughly checked the first draft and improved the first draft. All authors commented on previous versions of the manuscript. All three authors have read and approved the final manuscript.

## Conflict of Interest

The authors declare that the research was conducted in the absence of any commercial or financial relationships that could be construed as a potential conflict of interest.

## Publisher's Note

All claims expressed in this article are solely those of the authors and do not necessarily represent those of their affiliated organizations, or those of the publisher, the editors and the reviewers. Any product that may be evaluated in this article, or claim that may be made by its manufacturer, is not guaranteed or endorsed by the publisher.
